# The Effect of False-Positive Results on Subsequent Participation in Chest X-ray Screening for Lung Cancer

**DOI:** 10.2188/jea.JE20150106

**Published:** 2016-12-05

**Authors:** Akira Sato, Shota Hamada, Yuki Urashima, Shiro Tanaka, Hiroaki Okamoto, Koji Kawakami

**Affiliations:** 1 Department of Pharmacoepidemiology, Graduate School of Medicine and Public Health, Kyoto University, Kyoto, Japan; 2 Cancer Center, Yokohama Municipal Citizen’s Hospital, Yokohama, Japan; 3 Department of Respiratory Medicine, Yokohama Municipal Citizen’s Hospital, Yokohama, Japan

**Keywords:** screening, false positives, lung cancer, adherence, chest X-ray

## Abstract

**Background:**

High attendance rates and regular participation in disease screening programs are important contributors to program effectiveness. The objective of this study was to examine the effects of an initial false-positive result in chest X-ray screening for lung cancer on subsequent screening participation.

**Methods:**

This historical cohort study analyzed individuals who first participated in a lung cancer screening program conducted by Yokohama City between April 2007 and March 2011, and these participants were retrospectively tracked until March 2013. Subsequent screening participation was compared between participants with false-positive results and those with negative results in evaluation periods between 365 (for the primary outcome) and 730 days. The association of screening results with subsequent participation was evaluated using a generalized linear regression model, with adjustment for characteristics of patients and screening.

**Results:**

The proportions of subsequent screening participation within 365 days were 12.9% in 3132 participants with false-positive results and 6.7% in 15 737 participants with negative results. Although the differences in attendance rates were reduced with longer cutoffs, participants with false-positive results were consistently more likely to attend subsequent screening than patients with negative results (*P* < 0.01). The predictors of subsequent screening participation were false-positive results (risk ratio [RR] 1.72; 95% confidence interval [CI], 1.54–1.92), older age (RR 1.17; 95% CI, 1.11–1.23), male sex (RR 1.46; 95% CI, 1.29–1.64), being a current smoker (RR 0.80; 95% CI, 0.69–0.93), current employment (RR 0.79; 95% CI, 0.70–0.90), and being screened at a hospital cancer center (vs public health centers; RR 1.36; 95% CI, 1.15–1.60).

**Conclusions:**

Our findings indicated that subsequent participation in lung cancer screening was more likely among participants with false-positive results in an initial screening than patients with negative results.

## INTRODUCTION

Lung cancer is one of the leading causes of death from cancer for men and women in Japan.^1^ As patients with lung cancer are at risk of poor prognosis, early detection and treatment are important for successful disease management. Chest X-ray screening for lung cancer has been recommended by the Ministry of Health, Labour and Welfare and has been widely implemented by Japanese local governments^2^^,^^3^ based on the association of the screening with reductions in lung cancer mortality by approximately 30% to 60% from case-control studies performed in different regions of Japan.^4^^–^^10^

In Japan, attendance rates for various cancer screenings are generally low, and this has been recognized as an important issue.^11^ Low rates of initial participation and of re-attendance to screenings may compromise the effectiveness of screening programs. Studies conducted in breast cancer screening have shown that false-positive results in previous screenings can affect participation in subsequent screenings.^12^^–^^14^ In a systematic review of mammography screening for breast cancer, participants with false-positive results were found to be more likely to attend subsequent screenings in the United States but less likely in Europe and Canada.^14^ For lung cancer screening, false-positive results were identified as a negative predictor of subsequent screening attendance for participants in a randomized controlled trial, the Prostate, Lung, Colorectal, and Ovarian Cancer Screening Trial (PLCO).^15^ However, further confirmation of those findings are needed, since the behavior of participants in a screening trial may differ from that in actual clinical practice. In addition, false-positive results in screenings may engender unnecessary risks of physical or psychological complications associated with additional examinations.^12^^–^^14^^,^^16^ Therefore, the objective of this study was to examine the effects of initial false-positive results in chest X-ray screening for lung cancer on subsequent screening participation in a general community population.

## METHODS

### Lung cancer screening in Yokohama City

Since 1981, Yokohama City (with approximately 3.7 million residents) has conducted an annual lung cancer screening program using chest X-ray. The target subjects for screening comprised residents aged 40 years and older who did not have the opportunity to attend cancer screening at their workplace or who were unemployed residents. Participants with a smoking index of 400 or more were offered an optional sputum cytology test.

Screening institutions included the Cancer Center of Yokohama Municipal Citizen’s Hospital (CC-YMCH) and 18 Public Health and Welfare Centers (PHWC), which are located in each ward. In this study, we focused on the screening at these institutions provided by the local government of Yokohama City but did not include private complete medical check-ups and screenings conducted by other institutions. The screening fees (subsidized by Yokohama City) in 2014 were 680 or 1350 yen (approximately 6.8 or 13.5 United States dollars) for chest X-ray only or for both chest X-ray and sputum cytology test, respectively. These fees were waived for participants aged 70 years and older and for those who were from low-income families. In this way, Yokohama City supports candidate screening participants through subsidies to cover part or all of the screening costs.

**Figure 1.**
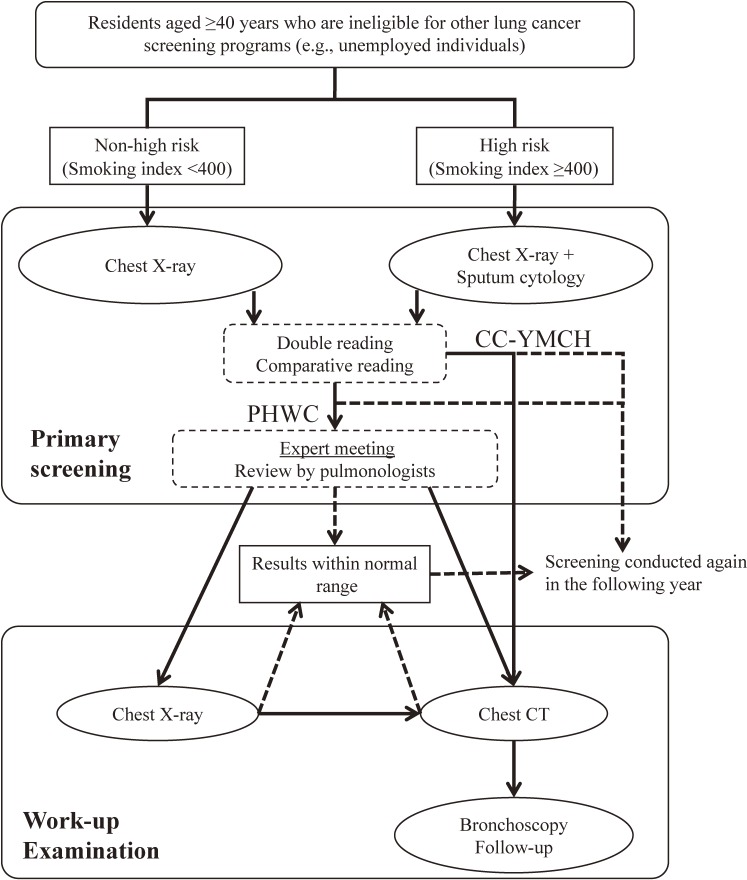
Flow diagram of the lung cancer screening process implemented by the local government of Yokohama City. In the primary screening, participants were examined by chest X-ray (indirect or direct radiograph), with or without additional sputum cytology test. There were two lung cancer screening systems: one was performed in the 18 Public Health and Welfare Centers (PHWC), and the other was in the Cancer Center of Yokohama Municipal Citizen’s Hospital (CC-YMCH). If participants had positive results (e1, e2, and d) in the primary screening, they were asked to undergo work-up examinations, such as chest X-ray (direct radiograph), chest computed tomography (CT), and bronchoscopy.

The flow of the lung cancer screening process in Yokohama City is shown in Figure 1. There were two lung cancer screening systems: one was conducted in the 18 PHWCs, and the other was conducted in the CC-YMCH. In the PHWC system, a public health physician and a primary care physician independently read chest radiographs (posterior-anterior and lateral views). Two pulmonologists at the CC-YMCH also independently read the same chest radiographs. These four physicians performed comparative readings of current and previous chest radiographs, if available. If any one of the four physicians detected an abnormality, the radiographs were reviewed in an expert meeting that involved pulmonologists from the CC-YMCH and physicians from the PHWCs, and the need for further investigations was determined. If participants initially received an indirect radiograph, further investigation included direct radiographs or chest computed tomography (CT) scans. If participants initially received direct radiographs, further investigation involved chest CT scans. In the CC-YMCH system for lung cancer screening, two pulmonologists independently read the direct radiographs. If at least one physician determined a positive result, further investigations were performed without an expert meeting.

Results of the screening were classified into five categories (“a” to “e”) according to guides published by the Japan Lung Cancer Society^2^^,^^17^^,^^18^; the categories of “suspicious (e2)” or “possible (e1)” were considered positive results. Suspicious noncancerous lesions that required further examination were categorized as “d”. Suspicious noncancerous lesions that did not require further examination were categorized as “c”. Normal chest radiographs were categorized as “b” and radiographs that were inadequate for reading were categorized as “a”.

Participants were informed of their screening results via mail. Participants with negative results were encouraged to attend screening 1 year later. Participants with positive results were asked to undergo work-up examinations by mail and/or telephone by public health nurses. As a result, almost all participants in the screening program with positive results (>97%) underwent work-up examinations. After screening, all screening records were collected and stored at the CC-YMCH.

### Study design and population

We conducted a historical cohort study to analyze participants aged 40–79 years who had attended lung cancer screening for the first time between April 2007 and March 2011 in Yokohama City. First-time participants were defined as those with no record of screening results from lung cancer screening conducted at a PHWC or the CC-YMCH within the preceding 5 years. Participants were excluded from analysis if they fulfilled any of the following criteria: participants who did not attend any work-up examinations; participants who had been diagnosed with lung cancer in the past; and participants with bloody sputum at the primary screening, as these participants were supposed to receive work-up examinations regardless of their chest X-ray screening results. Participants were tracked until March 2013, and, as a result, all participants were observed for a minimum of 2 years.

Prior to conducting this study, written informed consent was obtained from all screening participants for the use of their screening records for research purposes. The study protocol was approved (Approval Number E1884) by Kyoto University Graduate School and Faculty of Medicine Ethics Committee and the Institutional Review Board of Yokohama Municipal Citizen’s Hospital.

### Definitions

Primary screening included chest X-ray (indirect or direct radiograph) with or without additional sputum cytology. Work-up examinations included additional chest X-ray (direct radiograph), chest CT scan, and bronchoscopy. Participants were classified into false-positive, negative, and true-positive groups according to their screening results. Participants with either true-negative or false-negative results in the primary screening were collectively classified into the negative group, as we were unable to distinguish between these participants due to insufficient follow-up records. Due to the low prevalence of lung cancer in screened individuals, participants with false-negative results were expected to account for a very small proportion of the sample. The inclusion of these participants in the negative group is therefore unlikely to have a substantial impact on the results of the study.

Participants with false-positive results were defined as those who had positive results after the expert meeting in the primary screening but were not subsequently diagnosed with lung cancer. Participants without definitive diagnoses within 365 days of the first date of work-up examination visit were also included in the false-positive group. Participants with true-positive results were defined as those who were diagnosed with lung cancer within 365 days of the first date of work-up examination visit.

### Outcomes and measurements

The primary outcome measure was subsequent screening participation (screening adherence) within 365 days of determining false-positive or negative results. This study period was used because the efficacy of lung cancer screening has been shown to be higher in patients who had undergone screening within 365 days of case diagnosis.^10^

Measurements for this study included sex, age, smoking status, lung comorbidities, occupational history related to pneumoconiosis, family history of malignancy, current employment, screening institution, screening fees borne by participant, period of work-up examinations, and procedures of work-up examinations. Lung comorbidities included chronic obstructive pulmonary disease, bronchiectasis, interstitial pneumonia, asbestosis, sarcoidosis, and pneumoconiosis.

### Statistical analysis

We compared participants with false-positive results and those with negative results. Continuous variables were summarized as means and standard deviations, and categorical variables were summarized as proportions. Smoking status was categorized into three groups: non-smokers, who had never smoked cigarettes regularly; former smokers, who had previously smoked cigarettes regularly but were not current smokers; and current smokers, who smoked cigarettes regularly at the time of screening attendance. The proportions of subsequent screening participation were calculated. Student’s *t*-test and chi-square test were used to analyze continuous and categorical variables, respectively. Statistical significance level was set at 5% (two-sided).

Generalized linear regression with a log link, Poisson distribution, and robust error variances was used to compare the risk of subsequent participation with the screening results in the false-positive and negative results groups.^19^ Age was divided into 10-year intervals and the period of work-up examinations was divided into 30-day intervals before these variables were incorporated into the model. Categorical variables, except for smoking status, were treated as dichotomous variables. Measures of association were reported as risk ratios (RRs) with 95% confidence intervals (CIs). Covariates in the models were selected through univariate regression analysis using *P* values of less than 0.2 as the cutoff value; the final models included age, sex, smoking status, lung comorbidities, occupational history related to pneumoconiosis, family history of malignancy, current employment, screening institution, and screening fees. All potential explanatory variables were assessed for multicollinearity using the variance inflation factor (VIF), and variables were considered for omission if their VIF was greater than 5. The interaction between sex and smoking status was also tested in consideration of the relationship shown in a previous study.^15^

A sensitivity analysis was carried out by extending the duration of the period between primary screening (including work-up examinations) and subsequent screening participation to 455 days (1.25 years), 545 days (1.5 years), 635 days (1.75 years) and 730 days (2 years). A subgroup analysis for the false-positive results group was also conducted to assess the risk factors for subsequent participation. In the subgroup analysis, the period and procedures of work-up examinations were additionally used as covariates for adjustment. All analyses were performed using STATA version 13 (Stata corporation, College Station, TX, USA).

## RESULTS

### Demographic data and background characteristics

**Figure 2.**
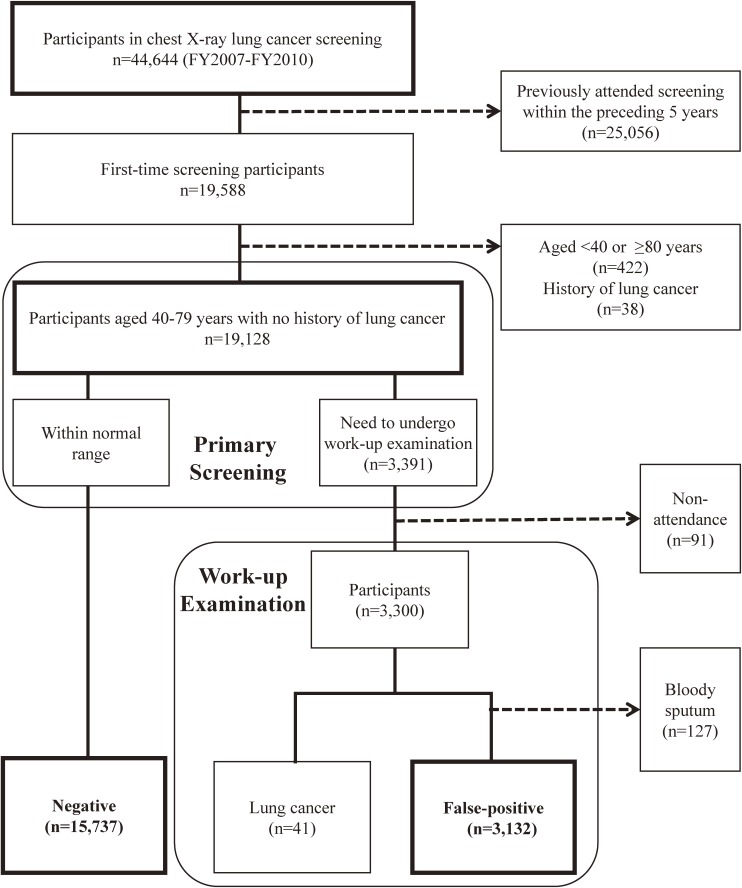
Selection of the study cohort. First-time lung cancer screening participants aged 40–79 years with no personal history of lung cancer, who had undergone work-up examinations, and who had no bloody sputum were included in the analysis. The study cohort consisted of 15 737 participants with negative results and 3132 participants with false-positive results. FY, fiscal year.

A total of 44 644 participants attended the chest X-ray screening for lung cancer provided by Yokohama City between April 2007 and March 2011. Among them, 19 588 (44%) were first-time screening participants, and 18 869 participants, including 15 737 (83%) with negative results and 3132 (17%) with false-positive results, were included in the analysis (Figure 2). As shown in Table 1, there were statistically significant differences between the negative and false-positive results groups in sex, age, smoking status, lung comorbidities, family history of malignancy, current employment, and screening fees (*P* < 0.05).

**Table 1.** Study cohort characteristics

Screening results	Overall	Negative	False Positive	*P* value
	(*n* = 18 869)	(*n* = 15 737)	(*n* = 3132)	
		
	*n* (%)	*n* (%)	*n* (%)	
Mean (SD) age, years	59.5 (10.8)	58.9 (10.9)	63.4 (9.6)	<0.01^a^
Sex				<0.01^b^
Female	11 244 (59.6)	9631 (61.2)	1613 (51.5)	
Male	7625 (40.4)	6106 (38.8)	1519 (48.5)	
Smoking status				<0.01^b^
Non-smoker	11 212 (59.4)	9429 (59.9)	1783 (56.9)	
Former smoker	3743 (19.8)	3042 (19.3)	701 (22.4)	
Current smoker	3914 (20.7)	3266 (20.8)	648 (20.7)	
Lung comorbidity				<0.01^b^
No	17 966 (95.2)	15 014 (95.4)	2952 (94.2)	
Yes	903 (4.8)	723 (4.6)	180 (5.7)	
Asthma	689 (3.7)	576 (3.7)	113 (3.6)	
COPD	138 (0.7)	99 (0.6)	39 (1.3)	
Bronchiectasis	85 (0.5)	60 (0.4)	25 (0.8)	
Interstitial pneumonia	9 (0.05)	3 (0.02)	6 (0.2)	
Asbestosis	4 (0.02)	2 (0.01)	2 (0.06)	
Sarcoidosis	10 (0.03)	3 (0.02)	3 (0.1)	
Pneumoconiosis	1 (0.01)	1 (0.01)	0 (0.0)	
Occupational history related to pneumoconiosis				0.17^b^
No	17 608 (93.3)	14 703 (93.4)	2905 (92.8)	
Yes	1261 (6.7)	1034 (6.6)	227 (7.2)	
Family history of malignancy				<0.01^b^
No	6452 (34.2)	5302 (33.7)	1150 (36.7)	
Yes (any type of malignancy)	12 417 (65.8)	10 435 (66.3)	1982 (63.3)	
Lung cancer	2466 (13.1)	2074 (13.2)	392 (12.5)	0.32^b^
Employment status				<0.01^b^
No	12 656 (67.1)	10 443 (66.4)	2213 (70.7)	
Yes	6213 (32.9)	5294 (33.6)	919 (29.3)	
Screened at CC-YMCH				0.11^b^
No	15 688 (83.1)	13 115 (83.3)	2573 (82.2)	
Yes	3181 (16.9)	2622 (16.7)	559 (17.8)	
Screening fees borne by participant				0.02^b^
No	1572 (8.3)	1278 (8.1)	294 (9.4)	
Yes	17 297 (91.7)	14 459 (91.9)	2838 (90.6)	
Procedures of work-up examinations				
No	—	—	40 (1.3)	
Chest X-ray (direct radiograph)	—	—	2363 (75.4)	
Chest computed tomography	—	—	1794 (57.3)	
Bronchoscopy	—	—	58 (1.9)	

CC-YMCH, Cancer Center of Yokohama Municipal Citizen’s Hospital; COPD, Chronic obstructive pulmonary disease; SD, standard deviation.

^a^Student’s *t*-test.

^b^Chi-square test.

There was no missing data for study cohort characteristics. Data are presented as *n* (%), unless otherwise noted.

### Subsequent screening participation

**Figure 3.**
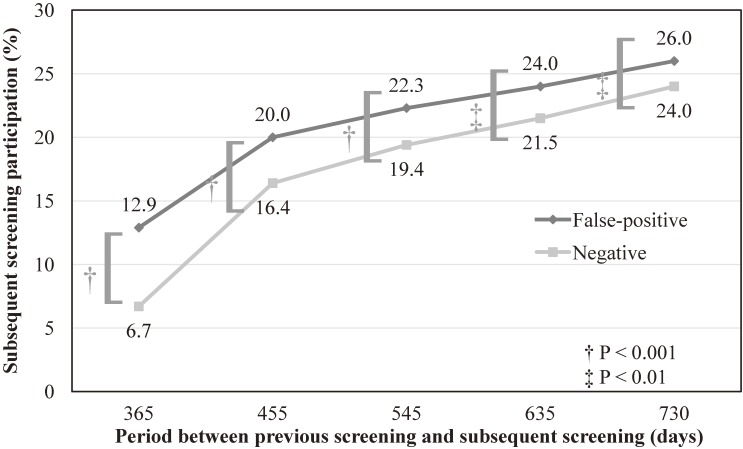
Sensitivity analysis of subsequent participation in lung cancer screening. The proportion of participants with subsequent screening participation was consistently higher in participants with false-positive results compared to those with negative results, even when the duration of the period between primary screening and subsequent screening was extended. However, the differences in proportions of participants with subsequent screening participation were reduced as longer durations were applied.

The proportions of subsequent screening participation were 6.7% and 12.9% in the negative and false-positive results groups, respectively. The crude risk difference (false-positive vs negative) was 0.06 (95% CI, 0.05–0.07), and the crude RR was 1.92 (95% CI, 1.73–2.14). In the sensitivity analysis (Figure 3), extending the period between the initial screening and subsequent screening resulted in reductions to the gaps in subsequent attendance rates between the negative and false-positive results groups; however, participants with false-positive results were still consistently more likely to attend subsequent screening (*P* < 0.01).

### Predictors of participation in subsequent screening

The multivariable analysis indicated that false-positive results (RR 1.72; 95% CI, 1.54–1.92), older age (RR 1.17; 95% CI, 1.11–1.23), male sex (RR 1.46; 95% CI, 1.29–1.64), being a current smoker (RR 0.80; 95% CI, 0.69–0.93), current employment (RR 0.79; 95% CI, 0.70–0.90), and being screened at the CC-YMCH (RR 1.36; 95% CI, 1.15–1.60) were statistically significant predictors of participation in subsequent screening (Table 2). Multicollinearity was not detected for the variables, and no interaction between sex and smoking status was observed.

**Table 2.** Predictors of participation in subsequent screening based on univariable and multivariable regression analysis for the overall study population

Variables	Reference	Univariable	Multivariable
	
		RR (95% CI)	RR (95% CI)
False-positive result	Negative result	1.92 (1.73–2.14)	1.72 (1.54–1.92)
Age (10-year increments)		1.28 (1.22–1.33)	1.17 (1.11–1.23)
Male	Female	1.42 (1.29–1.56)	1.46 (1.29–1.64)
Smoking status			
Former smoker	Non-smoker	1.09 (0.97–1.23)	0.90 (0.79–1.03)
Current smoker	Non-smoker	0.84 (0.73–0.95)	0.80 (0.69–0.93)
Lung comorbidity	None	0.91 (0.72–1.16)	0.95 (0.75–1.20)
Occupational history related to pneumoconiosis	None	0.88 (0.72–1.08)	0.82 (0.66–1.01)
Family history of malignancy	None	0.95 (0.86–1.05)	1.00 (0.90–1.11)
Current employment	None	0.73 (0.65–0.81)	0.79 (0.70–0.90)
Screened at CC-YMCH	Screened at PHWC	1.25 (1.11–1.41)	1.36 (1.15–1.60)
Screening fee borne by participant	Exempted	0.79 (0.68–0.93)	1.07 (0.86–1.33)

CC-YMCH, Cancer Center of Yokohama Municipal Citizen’s Hospital; CI, confidence interval; PHWC, Public Health and Welfare Center; RR, risk ratio.

### Subgroup analysis for participants with false-positive results

Table 3 shows the results of the subgroup analysis for participants with false-positive results. The results indicated that subsequent participation was positively associated with male sex (RR 1.34; 95% CI, 1.07–1.69) and being screened at the CC-YMCH (RR 1.80; 95% CI, 1.35–2.39) and negatively associated with being a current smoker (RR 0.72; 95% CI, 0.55–0.96) and current employment (RR 0.79; 95% CI, 0.62–0.99). These results were similar to the analysis for all participants. In addition, subsequent participation was positively associated with longer periods of work-up examinations (RR 1.14; 95% CI, 1.10–1.17).

**Table 3.** Predictors of participation in subsequent screening based on multivariable regression analysis for participants with false-positive results

Variables	Reference	RR (95% CI)
Age (10-year increments)		1.08 (0.97–1.21)
Male	Female	1.34 (1.07–1.69)
Smoking status		
Former smoker	Non-smoker	0.81 (0.62–1.04)
Current smoker	Non-smoker	0.72 (0.55–0.96)
Lung comorbidity	None	0.84 (0.54–1.29)
Occupational history related to pneumoconiosis	None	0.66 (0.44–1.01)
Family history of malignancy	None	0.98 (0.81–1.19)
Current employment	None	0.79 (0.62–0.99)
Screened at CC-YMCH	Screened at PHWC	1.80 (1.35–2.39)
Screening fee borne by participant	Exempted	0.98 (0.69–1.39)
Period of work-up examinations (30-day increments)		1.14 (1.10–1.17)
Work-up examinations: chest computed tomography and/or bronchoscopy	Chest X-ray (direct radiograph)	0.87 (0.71–1.06)

CC-YMCH, Cancer Center of Yokohama Municipal Citizen’s Hospital; CI, confidence interval; PHWC, Public Health and Welfare Center; RR, risk ratio.

## DISCUSSION

Attendance rates for lung cancer screening is low (about 20%) in Japan.^11^ While it is important to encourage participation in those who have never undergone lung cancer screening, it is also necessary to focus on screening adherence and encourage regular screening to maximize the benefits afforded by these programs. This study revealed a low re-attendance rate after the first-time screening for lung cancer in actual clinical practice and provides several suggestions to improve these rates. We demonstrated that participants with false-positive results were more likely to attend subsequent screening than participants with negative results. This may be partly explained by participants with false-positive results having more opportunities to be encouraged to attend subsequent screenings by medical professionals and to enhance their health awareness during their work-up examinations. On the other hand, participants with negative results are only encouraged to attend subsequent screenings during the primary screening. Therefore, a more proactive approach to ensure regular participation for those with negative results is needed. In contrast with the findings of our study, false-positive results were identified as a negative predictor of subsequent screening participation in PLCO study participants.^15^ We postulate that this disparity may be influenced by the underlying differences in participant characteristics (eg, fear of lung cancer and perceived risk due to radiation exposure) and awareness for cancer screening (eg, belief in screening effectiveness), as well as by different study designs, health insurance systems, and socio-psychological conditions.

We also identified other factors positively associated with participating in subsequent screening, including male sex, older age, screening institution, and a longer period of work-up examinations. Current smoking and current employment were negatively associated with subsequent screening participation. Current smokers may be less concerned about their health in general and therefore less likely to participate in subsequent screenings. A population-based approach to promote smoking cessation (eg, offering information on the merits of smoking cessation) may enhance screening participation. Employed people might think they are healthy or prioritize their work rather than participation in screenings. Improved access to screening (eg, building a remote diagnostic system) may be helpful for these people. Participants with longer periods of work-up examinations may have more opportunities to become familiar with the physicians and medical staff, so they may develop an increased awareness of their health.^12^ In contrast, CT scan and bronchoscopy may discourage participants from attending subsequent screenings, despite the lack of statistical significance in the present study, because these procedures place higher physical, psychological, and economic burdens on the examinees. These findings suggest the need for an individualized approach to facilitating regular screening participation according to characteristics of participants and screening methods. A theoretical approach based on behavioral economics and social marketing can be effectively applied to determine the individualized strategy to promote re-attendance to lung cancer screening, but further research will be needed.^20^^–^^22^

Studies have been conducted on the effects of false-positive results on breast cancer screening,^13^^,^^14^ which may give insight into the general underlying issues in cancer screening. A systematic review showed that participants with false-positive results in mammography for breast cancer had anxieties associated with the screening process.^14^ The participants with false-positive results in our study may also experience fear and anxiety associated with inspections that are more unpleasant or involve higher radiation exposure than the initial screening test, which may affect participation in subsequent screenings. In addition to such psychological considerations, screening intervals, target screening population, and the screening modalities may also affect screening participation behaviors. Therefore, it is necessary to evaluate the effectiveness of cancer screening in real clinical practice in the context of the various characteristics of screening. Previous studies of mammography screening indicate that differences in psychological backgrounds, social security systems, and national identity may affect screening participation.^12^^–^^14^

There are several limitations to this study. First, the participants included only those who did not have an opportunity to undergo cancer screening at their workplace and unemployed people. Thus, our study sample may not be representative of the entire population that is eligible for screening. In addition, the analysis may underestimate the rate of subsequent screening attendance because some participants may have joined other screening programs after the first-time screening. Second, we attempted to restrict our analysis to first-time screening participants with at least 5 years of non-attendance to the screening program. However, the sample may also include participants who have irregularly attended the screening program and had undergone screening more than 5 years prior. Third, this study could not provide insight into the reasons for failure to attend subsequent screening. Reasons for non-attendance may include death, re-location, and participation in other screening programs or medical check-ups. Psychological, economic, and other factors may also affect screening attendance, and we should consider these aspects when evaluating the effectiveness of the screening process. Fourth, Yokohama City is one of the most urbanized areas in Japan, and there may be difficulties in extrapolating our results to smaller, more rural areas due to differences in access to medical facilities. Finally, and possibly most importantly, we assumed the effectiveness of the screening test itself based only on evidence from case-control studies conducted in Japan,^5^^–^^10^ despite conflicting results from studies conducted in other countries. Chest X-ray screening for lung cancer has not been shown to reduce lung cancer mortality in the United States or Europe.^23^^–^^27^ There has yet to be a randomized controlled trial conducted in Japan to evaluate the effectiveness of chest X-ray screening for lung cancer.

In conclusion, this study demonstrated that subsequent participation in lung cancer screening in actual clinical practice was affected by the initial screening result and several patient characteristics. The predictors identified in this study may be useful for selecting the target population that should be actively encouraged to attend subsequent screenings. In Japan, there is a great need to improve cancer screening rates, and our findings may support the formulation of proactive measures to facilitate continuous and consistent participation in lung cancer screening.
